# Evolution of oncogenic signatures of mutation hotspots in tyrosine kinases supports the atavistic hypothesis of cancer

**DOI:** 10.1038/s41598-018-26653-5

**Published:** 2018-05-29

**Authors:** Weiran Chen, Yixue Li, Zhen Wang

**Affiliations:** 1 School of Life Science and Technology, Tongji University, Shanghai, China; 2 Key Lab of Computational Biology, CAS-MPG Partner Institute for Computational Biology, Shanghai Institutes for Biological Sciences, Chinese Academy of Sciences, Shanghai, China; 3 Shanghai Center for Bioinformation Technology, Shanghai Industrial Technology Institute, Shanghai, China; 4 Collaborative Innovation Center for Genetics and Development, Fudan University, Shanghai, China

## Abstract

Cancer has been shown as an evolutionary process emerging hallmarks that are reminiscent of unicellular organisms. Since cancer is mostly driven by somatic mutations, especially by oncogenic hotspot mutations, we proposed a molecular atavism of cancer caused by gain-of-function mutations in oncogenes. As tyrosine kinase (TK) family contains the largest subgroup of oncogenes with hotspot mutations, we traced the most predominant mutation hotspots of TK oncogenes across phylogeny with the domain information and adjacent sequences integrated as onco-signatures. We detected 9 out of 17 TK oncogenes with onco-homologs possessing an onco-signature, which could be divided into two classes by whether their onco-homologs existed in mammals or not. In Class I we identified mammalian onco-homologs assuming oncogenic functions with onco-signatures always intact in cancer, such as HCK and LYN. In Class II with no bona fide mammalian onco-homologs, Pyk2, a protist onco-homolog with an onco-signature of BRAF was found assuming oncogenic-like functions. Onco-signatures in both classes root deep in the primitive system. Together, these evidences supported our proposal that cancer can be driven by reverse evolution of oncogenes through gain-of-function mutations. And also for the first time, we provided the specific targets for experimental verification of the atavistic hypothesis of cancer.

## Introduction

More than one century ago, Boveri^1^ characterized the malignant tumour cell as a cell which lost properties that a normal tissue cell retained and tended to very primitive properties. As our knowledge of cancer extended largely after decades of extensive research, the essential element of Boveri’s idea was resurrected by the atavistic hypothesis of cancer^2,3^, which proposed that the evolutionary roots of cancer can be traced back to the early transitional phase from unicellularity(UC) to multicellularity(MC), before complex metazoans emerged about 600 million years ago. Evidence supporting this hypothesis has long remained observational until recently. A phylostratigraphic study suggested a link between cancer genes and the emergence of multicellular life^4^. By analyzing the expression profile of both a xenograft tumour at different stages^5^ and various tumor samples^6^, Chen *et al*. demonstrated an evolving convergence from multicellular state towards unicellular state both in cancer expression profile and functional status. A more recent study which incorporated phylostratigraphy into expression analysis revealed a shift to the preferential expression of genes originating from unicellular organisms in seven cancer types^7^. Although these evidences together demonstrated a general trend of atavism in tumorigenesis, the part unanswered with respect to the atavistic hypothesis is still large. In particular, whether cellular atavism from multicelluarity to unicellularity is the cause or the result of tumorigenesis remains elusive.

As cancer mostly arises from accumulation of somatic mutations with only a modest number of them conferring growth advantages^8^, uncovering the roles of the driver mutations in aspect of atavism may provide more direct evidence for the atavistic hypothesis. For example, Lu *et al*.^9^ conceived a possibility that, by devolving some functions that p53 acquired during its phylogenic evolution, missense mutations might transform p53 into an oncogenic state. To verify this ‘devolving-to-oncogene’ hypothesis, ancient homologs of p53 with oncogenic functions were needed, which may contain the feature that p53 acquired during tumorgenesis. Following this assumption, gain-of-function mutations occurring in the oncogenes would be more likely to restore the ancient function of corresponding homologs than those loss-of-function mutations occurring in the tumor suppressor genes. Here, gain-of-function mutation is a mutation that results in an increase in a gene’s activity or in acquiring a new molecular function or a new pattern of gene expression. In fact, large-scale genome sequencing of cancer found the mutational pattern of oncogenes were quite different from that of tumor suppressor genes^8^. In contrast to ubiquitous protein-truncating alterations of tumor suppressor genes, oncogenes tend to have missense mutations recurrently to occur at the same site. Considering the dominant gain-of-function roles of the recurrent mutations, we speculated that they would possibly drive the cancer process by mimicing the active forms of the ancient homologs.

To verify our hypothesis, we focused on the oncogenes with recurrent mutations in the tyrosine kinase (TK) domain. Firstly, TKs compose a major portion of oncogenes and the abnormal activation of a TK gene by somatic mutations often contributes to the uncontrolled proliferation of cells, the most fundamental hallmak of cancer. Secondly, TKs arose just before the emergence of Metazoa, which corresponds to the evolutionary roots hypothesized by the atavistic theory. Thirdly, the TK domain is extremely conserved in evolution, making their ancient homolog easily to be identified by sequence information. These facts collectively make TKs ideal subjects for studying the evolutionary origin of driver mutations in cancer. By mapping recurrent mutations in the TK domain along with the flanking sequences around them to their remote homologs, we found oncogene-like homologs across phylogeny, and some of them were reported to possess oncogenic roles.

## Results

### Detecting remote homologs of oncogenes based on onco-signatures

**Figure 1:**
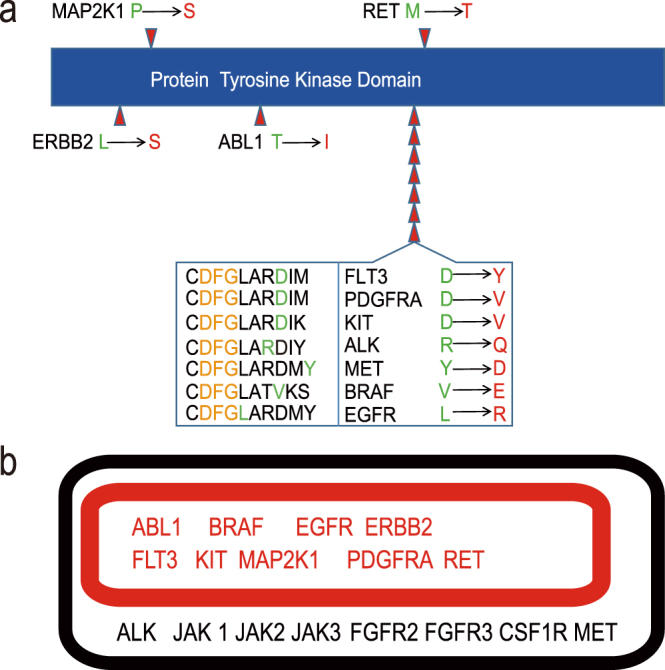
The 17 TK oncogenes with their dominant mutation hotspots. (**a**) The distribution of 11 dominant mutation hotspots in the TK domain. The red arrows point to the location, and the wild-type and mutant amino acids were indicated in green and red, respectively. (**b**) 9 out of the 17 oncogenes that passed the filter were shown in red box.

A total of 54 driver oncogenes^8^ with recurrent missense mutations were fetched from the COSMIC (Catalogue of Somatic Mutations in Cancer) database^10^. 17 of the oncogenes were assigned with a TK domain and we focused on the mostly mutated hotspots in the 17 genes (Supplementary Table S1). First we checked the mutation distribution of each hotspot in different tissue types. For each hotspot mutation, most of its occurrences fell into one particular tissue type except for BRAF V600E (Supplementary Table S2). Then we studied the positions of those hotspots in the TK domain. 11 out of the 17 mutation hotspots were located in the TK domain with an Asp-Phe-Gly (DFG)-motif, and 7 of them were closely adjacent to the DFG-motif (Fig. 1a). DFG-motif is a conserved motif that marks the beginning of the activation loop, and its conformation affects ATP substrate binding and catalytic competency of kinases^11^. As the mutation hotspots of TK oncogenes were prominently adjacent to the DFG-motif, this implied that the oncogenesis caused by TK mutations may largely result from the conformation changes related to the DFG-motif. In fact, several drugs such as dasatinib, nilotinib, vemurafenib and imatinib, target “in-or-out” conformations of the DFG-motif to treat cancer^11,12^. Besides the DFG-motif and the mutation hotspot itself, flanking sequences around the hotspot can also account for the oncogenic activation of TKs. For example, it was suggested that the BRAF Val600 mutation could mimic the phosphorylation of its adjacent site (Thr599/Ser602)^13^, which was required by Ras-mediated activation of the wild-type BRAF^14^.

Based on the knowledge above, we designed a signature-based mapping workflow to detect remote homologs of the TK oncogenes (Supplementary Fig. S1, see methods). Briefly, each recurrent mutation along with the flanking amino acids around it was treated as a signature (referred as “onco-signiture” in this study), which was then searched against the protein sequence database for cross-species homologs with a TK domain and a DFG-motif. The resulting homologs potentially preserved the oncogenic roles (referred as “onco-homolog”). Because fungi and plants have no orthodox TKs (although they have dual-specificity kinases) and TKs emerged as a late event just prior to the emergence of multicellular animals^15^, the search scope of species was subject to eukaryotes excluding fungi and plants.

We applied our methods to all the 17 TK oncogenes using data from Pfam^16^ database, and the onco-homologs of the 9 genes were identified (Fig. 1b and Supplementary Table S1). All the dominant hotspots of these 9 genes were on the TK domain containing a DFG-motif (Fig. 1a). To investigate the taxonomic distribution of the onco-homologs, we divided them into 6 taxonomic groups ranging from protists to mammals according to the NCBI genome annotation (Table 1 and Supplementary Table S3).

**Table 1:** Number of onco-homologs of the 9 TK oncogenes across taxonomic groups.

Gene	Protist	Flatworm	Roundworm	Insect	Fish	Reptile	Mammal	Class
ERBB2	5	0	5	0	0	0	0	I
MAP2K1	8	1	0	1	0	0	0	I
BRAF	7	0	9	1	1	0	1	I
FLT3	10	7	5	35	0	0	1	I
EGFR	3	0	4	0	2	0	2	I
ABL1	27	1	4	2	0	0	21	II
KIT	93	5	30	67	0	0	149	II
PDGFRA	89	4	28	67	0	0	149	II
RET	4	0	0	38	0	0	152	II

Based on the number of onco-homologs in mammals, we could split the 9 TK oncogenes into two classes. While genes in Class I including ABL1, KIT, PDGFRA and RET had considerable number of onco-homologs in mammals, those in Class II including ERBB2, MAP2K1, BRAF, FLT3 and EGFR had no or few onco-homologs in mammals. We manually checked the few mammalian onco-homologs in Class II and found they could probably result from sequencing errors (Supplementary Table S4). Since the onco-homologs in the two classes may exert potential oncogenenic effects in different taxonomic groups, we performed distinct analysis for each class, respectively.

### Class I onco-homologs in mammals represented by Src kinases

**Figure 2:**
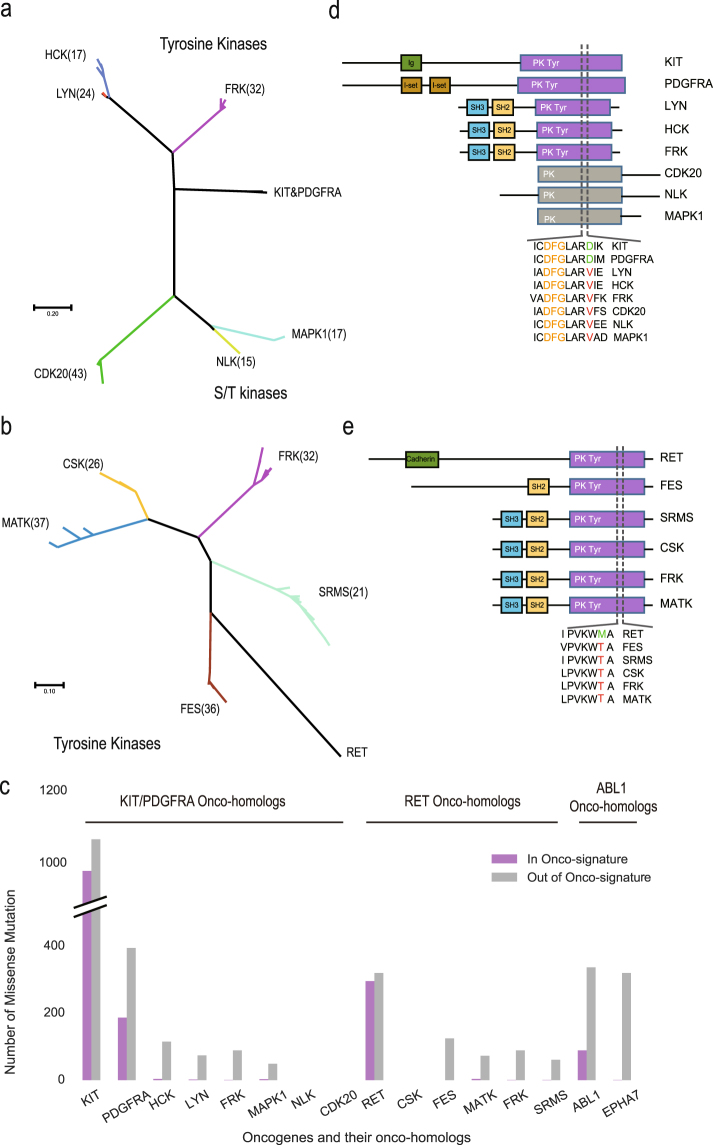
Human onco-homologs in Class I. (**a**,**b**) Phylogenetic trees of mammalian onco-homologs for (**a**) KIT/PDGFRA and (**b**) RET. As there was only one ortholog group (EPHA7) of onco-homologs for ABL1 in mammals, its tree was not shown here. (**c**) Comparison of mutation frequency for oncogenes and their onco-homologs. Purple and grey bars indicated the mutations in and out of onco-signatures, respectively. (**d**,**e**) Domain organizations and onco-signatures of human onco-homologs for (**d**) KIT/PDGFRA and (**e**) RET. Green and red letters indicated wild-type and mutant amino acids in the oncogene, respectively.

As for Class I, our results indicated the existence of TK oncogenes and their onco-homologs in mammalian genomes simultaneously. To investigate which protein family these onco-homologs belonged to, we aligned the protein sequences and reconstructed the phylogenetic tree for each oncogene and all its mammalian onco-homologs (Fig. 2a,b). We found that most of the onco-homologs could be represented by their human orthologs. For example, the mammalian onco-homologs of KIT and PDGFRA were clustered into 6 ortholog groups (Fig. 2a), where HCK, LYN and FRK were tyrosine kinases while NLK, MAPK1 and CDK20 were serine/theronine kinases. The mammalian onco-homologs of RET could be ascribed to 5 human ortholog groups named CSK, FRK, SRMS, MATK and FES (Fig. 2b), and the mammalian onco-homologs of ABL1 could be ascribed to EPHA7, all of which belonged to TKs.

To evaluate the oncogenic potential of the onco-homologs, we first searched the functional annotations in Uniprot database^17^. We found three of the onco-homologs (HCK, LYN and FES) were known as proto-oncogenes, which were invoked by aberrant gene expression. Besides, in contrast to KIT, PDGFRA and RET which contained high frequency of somatic mutations, the mutation frequency in the three onco-homologs were much lower (Fig. 2c). These results were consistent with our hypothesis because if the onco-signature could stand for the oncogenic functions, the onco-homologs could develop the functions by increased expression without somatic mutations. To investigate this in more detail, we compared the number of somatic mutations in and out of the onco-signature (Fig. 2c). As expected, while somatic mutations in KIT, PDGFRA, RET and ABL1 were prone to happen at the position within the range of onco-signature, their onco-homologs had few such mutations.

The functional relevance between the receptor TK KIT/PDGFRA and their onco-homologs, which shared the same onco-signature (Fig. 2d), was particularly remarkable. It was known that the mutation hotspot of KIT (D816V) and PDGFRA(D842V) were mutually exclusive and shared similar oncogenic mechanism in gastrointestinal stromal tumours (GISTs)^18^. Among their onco-homologs, HCK, LYN and FRK were members of the Src family, which were the downstream kinases normally activated by the wild-type KIT or PDGFRA^19^. In cancer cells, instead of performing the Src family kinase activation, the KIT D816V mutant gained a Src-like kinase activity itself, which then circumvented the need of downstream Src family kinases^20^. In fact, KIT, PDGFRA, LYN, HCK, FRK were all among the most sensitive targets of the same kinase inhibitor named dasatinib^21^. Besides, as another onco-homolog of KIT/PDGFRA, MAPK1 was a member of the RAS/ERK signalling pathway, which was also a downstream signaling pathway activated by KIT/PDGFRA. Though there were fewer researches compared with KIT/PDGFRA, RET was also a TK receptor with Src family kinases as its downstream signaling components^22^. Not surprisingly, the onco-homologs of RET included 2 Src-family kinases (CSK, FRK) and 2 Src-related kinases (SRMS, MATK) (Fig. 2e). All of the onco-homologs contained the SH2 domain (Fig. 2d,e), indicating that RET might share the same oncogenic mechanism with KIT/PDGFRA. Taken together, these results suggested a common mechanism that for Class I, the oncogene with mutation hotspots might acquire the oncogenic functions by mimicking its onco-homologs in the same signaling pathway.

### Oncogenic-like function of Class II onco-homologs in remote species

Unlike Class I, the oncogenes in Class II had no mammalian onco-homologs, indicating strong purifying selection for the onco-signatures in mammals. So we turned to search the onco-homologs within more distant taxonomic groups. Although a handful of onco-homologs for all of the five Class II oncogenes have been identified in distant species, it was not clear whether the onco-signatures in these species could be explained by oncogenic functions or other random events such as neutral substitutions and sequencing errors. To distinguish the two possibilities, we designed a statistical test to evaluate the significance of the onco-homolog number in remote taxonomic groups. Briefly, we took mutations occurring only once in the same oncogene from COSMIC as background, and searched for distant ‘homologs’ based on their sequence signatures as we did for the mutation hotspot (Supplementary Dataset S1). sSimilar to the hotspots in Class II, only mutations without mammalian ‘homologs’ were retained. As most of the background mutations were random in tumorigenesis, the number of such ‘homologs’ in each remote taxonomic group could be treated as the null distribution, which was used to evaluate the empirical p-value of the onco-homolog number (Supplementary Fig. S2).

**Figure 3:**
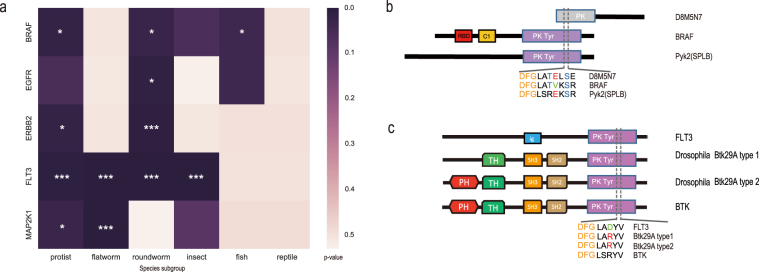
Onco-homologs in Class II. (**a**) Over-representation of onco-homologs across non-mammalian organism groups. The heatmap indicated statistical significance in the number between onco-homologs and random-site-extracted homologs (*P < 0.05, ***p < 0.01). (**b**) Protist onco-homolog Pyk2, D8M5N7aligned with its proto-oncogene BRAF. Green and red letter represented the wild-type and mutant amino acids in BRAF, respectively. (**c**) Domain organizations and onco-signatures of FLT3, isoforms of onco-homologs Btk29 in fly and human BTK.

In protists, we found that the numbers of onco-homologs for most Class II oncogenes were statistically significant (empirical p-value < 0.05, Fig. 3a), indicating a universal over-representation of the onco-signatures at the emergence of the TK signalling system. Since the onco-signatures could represent the oncogenic activation of TKs, this result suggested that the remote onco-homologs might help to adapt a primitive life. Although the functions of most onco-holomogs were poorly characterized in protists, we detected a protist onco-homolog of BRAF, Pyk2 (splB) with validated oncogenic-like function. *Dictyostelium* Pyk2 had the highest identity score with BRAF among all the protist onco-homologs by Blastp (Supplementary Table S5). It is a TK-like kinase which was intermediate between TKs and serine/threonine kinases. In the onco-signature, it contained a serine exactly in the same position as the activiting Ser602 in the BRAF V600E mutant (Fig. 3b). It was reported that Pyk2 could constitutively activate STATc, a Dictyostelium homolog of human STAT5. Considering that the STAT proteins play a pivotal role in TK signalling pathways and persistent activation of them were found in a variety of cancer types^23^, it is reasonable to characterize the function of Pyk2 as an ancient oncogene. We also found another uncharacterized onco-homolog (UniProtKB: D8M5N7) of BRAF in Blastocystis hominis with strong onco-signature containing Ser602 and Thr599, which was critical for V600E BRAF activation. And its oncogenic potential was worth further investigation.

The onco-homologs in other taxonomic groups could also show statistical significance (Fig. 3a), where an interesting example was Src-like kinase Btk29A, an insect onco-homolog of FLT3. There were two isoforms of Drosophila Btk29A. The type 2 isoform had one more domain than the type 1, and both of them carried the onco-signature DFGLARV (Fig. 3c). It was reported that mutants containing the type 1 isoform but not type 2 isoform could cause ovarian tumor in fly, while overexpression of the type 2 isoform could rescue the defection^24,25^. In humans, the BTK protein displayed DFGLSRV sequence in the position of FLT3 onco-signature with the similar domain organization to the type 2 isoform, but the type 1 isoform was lacking. Therefore, the FLT3 onco-homologs formed a counterbalancing mechanism only in insects, which may explain why the onco-signatures of FLT3 could so predominantly exist in insects.

## Discussion

Previous studies have shown the multicellularity-to-unicellularity pattern in cancer and ascribed this atavism to loss-of-function effects disrupting the multicellularity-supporting system [5–7]. However, gain-of-function variants such as hotspot mutations in oncogenes haven’t been considered. Given the oncogenic activity conferred by the gain-of-function variants and their prominent roles in tumorigenesis, we proposed another form of cancer atavism which caused by reverse evolution of oncogene itself through a gain-of-function way. Unlike transcriptome-wide evidences provided by previous studies, this form of atavism could be a driving force rather than an accompanying incident in cancer development.

We illustrated the atavism of oncogenes using the TK family. Proteins in the TK family function in transmembrane signaling as well as in intracellular signal transduction^26^. The TK domain was quite conserved in evolution and its emergence was accompanied by the evolutionary transition from unicellularity to multicellularity^15,27^, which means that TKs were widely spread in both forms of lives and involved in the formation of multicellular lives. In cancers, TKs have been found with a large group of recurrent oncogenic mutations conferring constitutive activation rather than inactivation of translated proteins. The facts above left TK’s role in tumorgenesis appropriately to be understood within the atavistic hypothesis of a multicellularity-to-unicellularity transition driven by gain-of-function mechanism.

To prove our hypothesis, we looked into the most frequently mutated sites in the oncogenes with a TK domain and traced the origin of the mutations across phylogeny (Fig. 1a). We found over half of the mutations happened within the TK domain, especially near the DFG-motif. Different kinases like FLT3, KIT and PDGFRA even shared the same mutation site and adjacent sequence. This result suggested that the oncogenic roles of those powerful mutations were highly dependent on the domain context, despite the divergence among TK genes. Thus, we used the mutation sites and adjacent sequences along with their domain context to search for the homologs that were potentially oncogenic. Compared with previous studies based on Blastp between protein sequences, our domain-centered method was more sensitive and efficient to explore the most promising candidates, especially for distant homologs.

Of all the onco-homologs, there were hundreds forming ortholog groups in mammals (Fig. 2a,b), and their oncogenes were categorized as Class I. A plausible explanation was that these mammalian onco-homologs may have potential oncogenic functions as the mutated oncogenes. For example, Src family kinases HCK, FRK and LYN are onco-homologs of PDGFRA/KIT, which interact with both PDGFRA and KIT as downstream effectors and play critical roles in tumor cell proliferation and survival^28^. Although there were few somatic mutations of the onco-homologs especially in the onco-signatures, some of them were well recognized as proto-oncogenes by over-expression. For instance, over-expressing of LYN leads to B-cell chronic lymphocytic leukemia^29^. Moreover, it was reported that the KIT D816V mutant could activate the downstream pathway independent of the Src family kinases, which implied that the mutated oncogenes in Class I could mimic the function of their onco-homologs. In concordance with this, the KIT D816V mutant also shared the same inhibitor against cancer with HCK and LYN^30^. Interestingly, all the genes in Class I are among the target of Imatinib, though the situation for particular mutants could be complex.

Our results also suggested a deep evolutionary root of oncogenic mutations in TKs (Table 1). Although no confident onco-homologs in mammals could be identified for Class II, the onco-signatures were significantly enriched in protists compared with signatures of random mutations (Fig. 3a), which implied their functional potentials in protists. Notably, as an onco-homolog of BRAF in *Dictyostelium discoideum*, Pyk2 was verified as a direct and constitutive activator of STATc^31^, which was a close ortholog of the metazoan STATs^32^. Considering that *Dictyostelium discoideum* has a multicellular form, this result supported the atavistic hypothesis proposed by Davies *et al*.^2^ that cancer could arise by unleashing the suppressed Metazoa 1.0 system.

## Methods

### Onco-homolog detection

The list of oncogenes with recurrent mutations was obtained from a previous study^8^. For each TK oncogene, the mutation hotspot with the highest frequency in cancers was extracted from COSMIC^10^. The mutated amino acid at the hotspot along with its flanking sequences were extracted as the “onco-signature”, with the length ranging from 3 to 8 amino acids at either side of the position. Firstly, the Pfam^16^ sequence database was searched by phmmer in the HMMER^33^ v3.1b to get all homologous proteins for each TK oncogene and their sequences were retrieved from Uniprot^17^ Ref 100. Next, against the retrieved homologous sequences we searched for onco-homologs using corresponding onco-signatures. The homologs with an onco-signature were aligned with its TK oncogene by hmmalign in HMMER, and only those with the onco-signature at the same position in the TK domain as the oncogene were preserved. At last, given that over half of the hotspots in the oncogenes were DFG-related, homologous sequences without the DFG-motif were excluded. Among the protist onco-homologs, we also aligned against its oncogene using blastp^34^ to find the one with the highest identity score.

### Gene tree

Multiple sequence alignment of mammalian onco-homologs for the Class I oncogenes were generated by Clustal Omega^35^. Then Mega7^36^ was used to construct the maximum likelihood tree of the onco-homologs.

### Somatic mutations in onco-homologs

For the Class I oncogenes and their onco-homologs in the human, all the somatic mutations were obtained from COSMIC. Mutations that happened within the onco-signature were defined as the “signature-in” mutation. The onco-signatures used here were those shared by the oncogene and its human onco-homologs.

### Statistical test of onco-homolog number in non-mammals

For TK oncogenes in Class II, we performed the same analysis for the mutation sites that occurred only once according to COSMIC as we did for the mutation hotspots. The mutated amino acid along with its flanking 3–8 amino acids at either side were treated as signatures of the background, which were then used to retrieve ‘homologs’ based on their alignment of the TK domain. As the mutation hotpots in Class II, we excluded the random sites (sites mutated once in COSMIC) with any mammalian ‘homologs’ detected. The empirical null distribution was obtained from the number of ‘homologs’ in each taxonomic group. The empirical p-value for the number of onco-homologs in each group was based on the null distribution. If the empirical p-value was less than 0.05, the null hypothesis was rejected and the onco-homologs were over-represented in the taxonomic group.

## Electronic supplementary material

Supplementary information

Dataset S1

## References

[1] Boveri T (2008). Concerning the Origin of Malignant Tumours byTheodor Boveri. Translated and annotated by Henry Harris. Journal of Cell Science.

[2] Davies PC, Lineweaver CH (2011). Cancer tumors as Metazoa 1.0: tapping genes of ancient ancestors. Physical Biology.

[3] Vincent M (2012). Cancer: a de-repression of a default survival program common to all cells?: a life-history perspective on the nature of cancer. Bioessays News & Reviews in Molecular Cellular & Developmental Biology.

[4] Domazet-Lošo T, Tautz D (2010). Phylostratigraphic tracking of cancer genes suggests a link to the emergence of multicellularity in metazoa. Bmc Biology.

[5] Chen H, Lin F, Xing K, He X (2015). Corrigendum: The reverse evolution from multicellularity to unicellularity during carcinogenesis. Nature Communications.

[6] Chen H, He X (2016). The Convergent Cancer Evolution toward a Single Cellular Destination. Molecular Biology & Evolution.

[7] Trigos AS, Pearson RB, Papenfuss AT, Goode DL (2017). Altered interactions between unicellular and multicellular genes drive hallmarks of transformation in a diverse range of solid tumors. Proceedings of the National Academy of Sciences of the United States of America.

[8] Vogelstein B (2013). Cancer Genome Landscapes. Science.

[9] Lu WJ, Amatruda JF, Abrams JM (2009). p53 ancestry: gazing through an evolutionary lens. Nature Reviews Cancer.

[10] Forbes SA (2017). COSMIC: somatic cancer genetics at high-resolution. Nucleic Acids Research.

[11] Bollag G (2012). Vemurafenib: the first drug approved for BRAF-mutant cancer. Nature Reviews Drug Discovery.

[12] Zhang J, Yang PL, Gray NS (2009). Targeting cancer with small molecule kinase inhibitors. Nature Reviews Cancer.

[13] Davies H (2002). Mutations of the BRAF gene in human cancer. Nature.

[14] Zhang BH, Guan KL (2000). Activation of B-Raf kinase requires phosphorylation of the conserved residues Thr598 and Ser601. Embo Journal.

[15] Lim WA, Pawson T (2010). Phosphotyrosine Signaling: Evolving a New Cellular Communication System. Cell.

[16] Finn RD (2016). The Pfam protein families database: towards a more sustainable future. Nucleic Acids Research.

[17] Apweiler R (2004). UniProt: the universal protein knowledgebase. Nucleic acids research.

[18] Martínbroto J, Rubio L, Alemany R, Lópezguerrero JA (2010). Clinical implications of KIT and PDGFRA genotyping in GIST. Clinical & Translational Oncology.

[19] Lennartsson J, Jelacic T, Linnekin D, Shivakrupa R (2005). Normal and oncogenic forms of the receptor tyrosine kinase kit. Stem Cells.

[20] Sun J, Pedersen M, Rönnstrand L (2009). The D816V mutation of c-Kit circumvents a requirement for Src family kinases in c-Kit signal transduction. Journal of Biological Chemistry.

[21] Montero JC, Seoane S, Ocaña A, Pandiella A (2011). Inhibition of SRC family kinases and receptor tyrosine kinases by dasatinib: possible combinations in solid tumors. Clinical Cancer Research An Official Journal of the American Association for Cancer Research.

[22] Plazamenacho I, Mologni L, Mcdonald NQ (2014). Mechanisms of RET signaling in cancer: current and future implications for targeted therapy. Cellular Signalling.

[23] Yu H, Jove R (2004). The STATs of cancer–new molecular targets come of age. Nature Reviews Cancer.

[24] Hamada-Kawaguchi N, Yamamoto D (2014). Btk29A promotes Wnt4 signaling in the niche to terminate germ cell proliferation in Drosophila. Science.

[25] Hamada‐Kawaguchi, N. & Yamamoto, D. Ovarian polarity and cell shape determination by Btk29A in Drosophila. *Genesis* (2017). 10.1002/dvg.23042 28639397

[26] Lemmon MA, Schlessinger J (2010). Cell signaling by receptor tyrosine kinases. Cell.

[27] Chiassonmackenzie, C. & Mcclatchey, A. I. Cell-Cell Contact and Receptor Tyrosine Kinase Signaling. *Cold Spring Harbor Perspectives in Biology*, a029215 (2017). 10.1101/cshperspect.a029215 PMC5983194 28716887

[28] Zhang S, Yu D (2012). Targeting Src family kinases in anti-cancer therapies: turning promise into triumph. Trends in Pharmacological Sciences.

[29] Tibaldi E (2011). Lyn-mediated SHP-1 recruitment to CD5 contributes to resistance to apoptosis of B-cell chronic lymphocytic leukemia cells. Leukemia.

[30] Schittenhelm MM (2006). Dasatinib (BMS-354825), a dual SRC/ABL kinase inhibitor, inhibits the kinase activity of wild-type, juxtamembrane, and activation loop mutant KIT isoforms associated with human malignancies. Cancer research.

[31] Araki T, Kawata T, Williams JG (2012). Identification of the kinase that activates a nonmetazoan STAT gives insights into the evolution of phosphotyrosine-SH2 domain signaling. Proceedings of the National Academy of Sciences of the United States of America.

[32] Araki T, Langenick J, Gamper M, Firtel RA, Williams JG (2008). Evidence that DIF-1 and hyper-osmotic stress activate a Dictyostelium STAT by inhibiting a specific protein tyrosine phosphatase. Development.

[33] Eddy SR (1998). Profile hidden Markov models. Bioinformatics (Oxford, England).

[34] Altschul SF (1997). Gapped BLAST and PSI-BLAST: a new generation of protein database search programs. Nucleic acids research.

[35] Sievers F (2011). Fast, scalable generation of high‐quality protein multiple sequence alignments using Clustal Omega. Molecular systems biology.

[36] Kumar S, Stecher G, Tamura K (2016). MEGA7: Molecular Evolutionary Genetics Analysis version 7.0 for bigger datasets. Molecular biology and evolution.

