# Supplementary material for: Evolution of oncogenic signatures of mutation hotspots in tyrosine kinases supports the atavistic hypothesis of cancer
Source: Sci Rep. 2018 May 29;8:8256. doi: 10.1038/s41598-018-26653-5 (PMC5974376; doi:10.1038/s41598-018-26653-5)
Supplement: Supplementary file 1 — Supplementary information [file 41598_2018_26653_MOESM1_ESM.docx]

**Supplementary Information**

**Evolution of oncogenic signatures of mutation hotspots in tyrosine kinases supports the atavistic hypothesis of cancer**

Weiran Chen^1^, Yixue Li^2,3,4^*, Zhen Wang^2^*

^1^School of Life Science and Technology, Tongji University, Shanghai, China;^2^Key Lab of Computational Biology, CAS-MPG Partner Institute for Computational Biology, Shanghai Institutes for Biological Sciences, Chinese Academy of Sciences, Shanghai, China,^3^Shanghai Center for Bioinformation Technology, Shanghai Industrial Technology Institute, Shanghai, China,^4^Collaborative Innovation Center for Genetics and Development, Fudan University, Shanghai, China.

*Corresponding author E-mail: yxli@sibs.ac.cn (Y. Li); zwang01@sibs.ac.cn (Z. Wang).

**
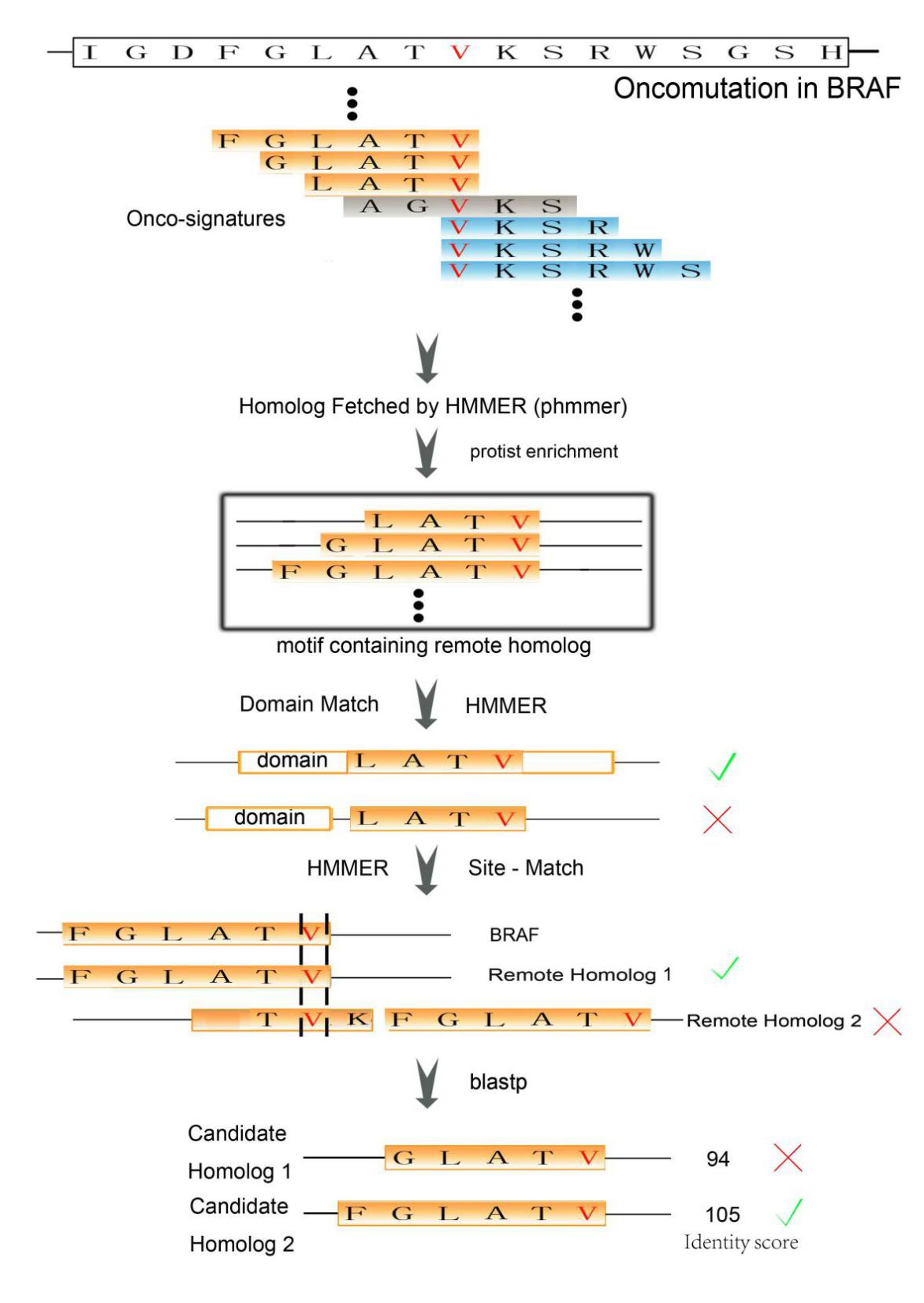
**

**Supplementary Fig. S1. The workflow of the onco-homolog detection.** Homolog sequence id were acquired from Pfam database ^1^. HMMER v3.1b(<http://hmmer.org/>) was used for ancient homolog detection and onco-signature mapping. Homolog sequences were retrieved from Uniprot^2^ ref 100. At last, Blastp ^3^ was used for best candidate homolog detection .

**
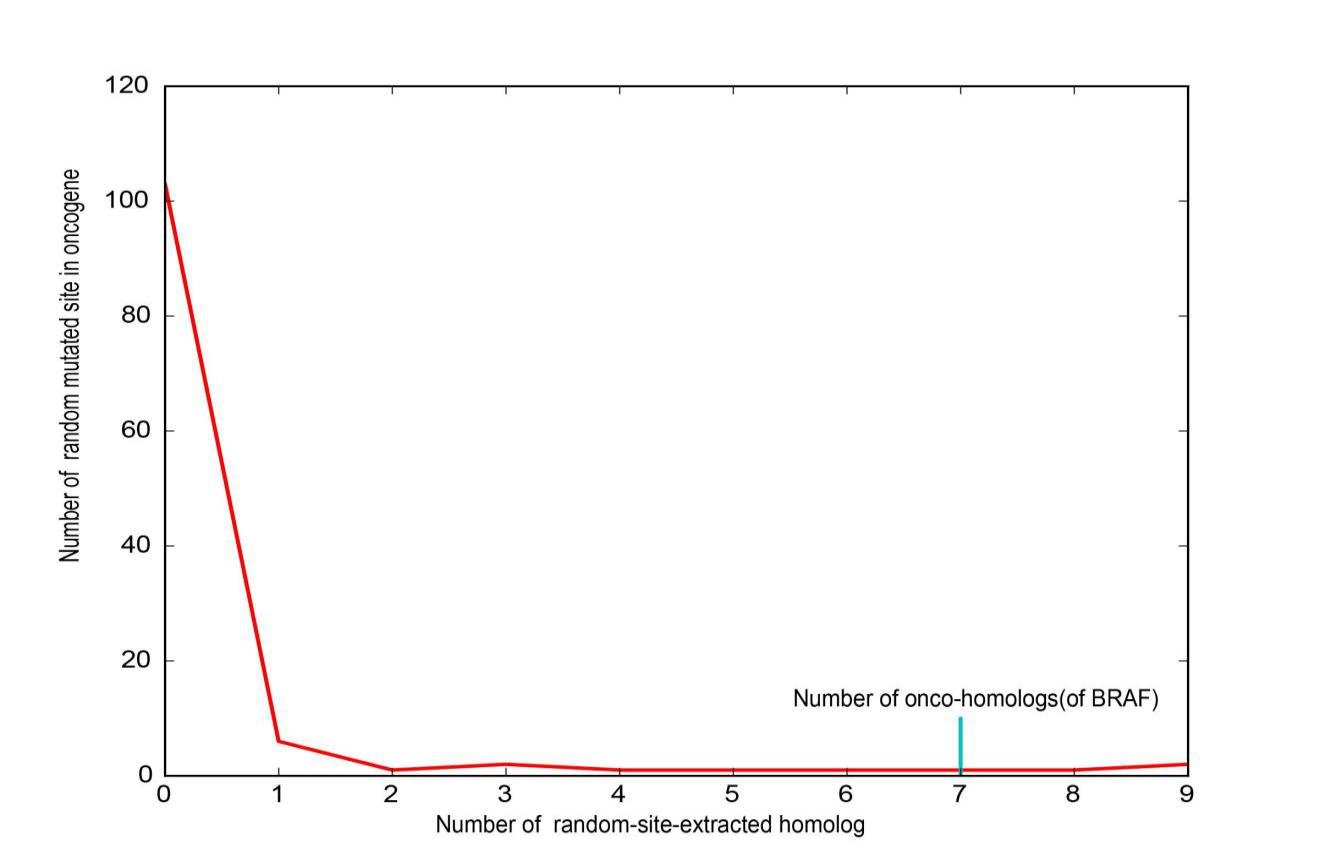
**

**Supplementary Fig. S2. The null distribution used to calculate the empirical p-value for the onco-homologs of BRAF.**

**Supplementary Table S1. The mutation hotspots used for onco-homolog searching and the searching results.**

| Gene | Mutation hotspots | Onco-homolog searching results ^a^ | |
| --- | --- | --- | --- |
| ABL1 | p.T315I | | Positive |
| ALK | p.R1275Q | | Negative (No DFG Motif) |
| BRAF | p.V600E | | Positive |
| CSF1R | p.Y969C | | Negative (Onco-motif alignment failed) |
| EGFR | p.L858R | | Positive |
| ERBB2 | p.L755S | | Positive |
| FGFR2 | p.S252W | | Negative (Onco-motif alignment failed) |
| FGFR3 | p.S249C | | Negative (Onco-motif alignment failed) |
| FLT3 | p.D835Y | | Positive |
| JAK1 | p.R724H | | Negative (Onco-motif alignment failed) |
| JAK2 | p.V617F | | Negative (Onco-motif alignment failed) |
| JAK3 | p.R657Q | | Negative (Onco-motif alignment failed) |
| KIT | p.D816V | | Positive |
| MAP2K1 | p.P124S | | Positive |
| MET | p.Y1235D(p.Y1253d) | | Negative (Onco-motif alignment failed) |
| PDGFRA | p.D842V | | Positive |
| RET | p.M918T | | Positive |

^a^ “Positive” and “negative” indicate onco-homologs were and were not found, respectively.

**Supplementary Table S2. Distribution of recurrent mutations in different** **tissue types(Top3).**

| Hotspots | Adrenal gland | Breast | Haematopoietic  and lymphoid | Large intestine | Lung | NS | Oesophagus | Skin | Small intestine | Solft tissue | Stomach | Testis | Thyroid | Urinary |
| --- | --- | --- | --- | --- | --- | --- | --- | --- | --- | --- | --- | --- | --- | --- |
| ABL1 p.T315I |  |  | 320 |  |  |  |  | 3 |  |  |  |  |  |  |
| BRAF p.V600E |  |  |  | 10533 |  |  |  | 9240 |  |  |  |  | 23922 |  |
| EGFR p.L858R |  | 15 |  |  | 10028 |  | 14 |  |  |  |  |  |  |  |
| ERBB2 p.L755S |  | 28 |  |  |  |  |  |  | 6 |  | 5 |  |  |  |
| FLT3  p.D835Y |  |  | 79 |  |  |  |  |  |  |  |  |  |  |  |
| KIT  p.D816V |  |  | 1586 |  |  |  |  | 8 |  |  |  | 17 |  |  |
| MAP2K1 p.P124S |  |  |  |  |  | 3 |  | 32 |  |  |  |  |  | 2 |
| PDGFRA p.D842V |  |  |  | 6 |  |  |  |  |  | 540 | 15 |  |  |  |
| RET  p.M918T | 31 | 1 |  |  |  |  |  |  |  |  |  |  | 727 |  |

**Supplementary Table S3. List of all identified onco-homologs.**

| Gene | Onco-Signature | Onco-homolog (UniProtKB) | Taxonomy |
| --- | --- | --- | --- |
| BRAF | DFGLATE | I3KH39 | fishes |
| EGFR | DFGR | H2SGY4 | fishes |
| EGFR | DFGR | H2SGY5 | fishes |
| ABL1 | IEFM | G4LYL1 | flatworms |
| FLT3 | DFGLARY | D1H0Y7 | flatworms |
| FLT3 | DFGLARY | G4VGW8 | flatworms |
| FLT3 | DFGLARY | G7YHQ9 | flatworms |
| FLT3 | DFGLARY | I4ENX0 | flatworms |
| FLT3 | DFGLARY | I4ENX1 | flatworms |
| FLT3 | DFGLARY | W6UV78 | flatworms |
| FLT3 | DFGLARY | W6V4U1 | flatworms |
| PDGFRA | DFGLARV | G4V8J8 | flatworms |
| KIT | DFGLARV | H2KTZ6 | flatworms |
| PDGFRA | DFGLARV | Q5D8E8 | flatworms |
| PDGFRA | ICDFGLARV | Q6PMM5 | flatworms |
| PDGFRA | DFGLARV | W6UZ63 | flatworms |
| MAP2K1 | SYIV | C4Q5Z0 | flatworms |
| ABL1 | YIII | E0VX53 | insects |
| ABL1 | YIII | T1HC48 | insects |
| BRAF | DFGLATE | W5JQH6 | insects |
| FLT3 | DFGLARY | A0A026WCK3 | insects |
| FLT3 | DFGLARY | A0A034W621 | insects |
| FLT3 | DFGLARY | A0A034W9Q3 | insects |
| FLT3 | DFGLARY | B0W172 | insects |
| FLT3 | DFGLARY | B0W3P6 | insects |
| FLT3 | DFGLARY | B3MPH0 | insects |
| FLT3 | DFGLARY | B3N732 | insects |
| FLT3 | DFGLARY | B4G907 | insects |
| FLT3 | DFGLARY | B4HYH7 | insects |
| FLT3 | DFGLARY | B4JBV4 | insects |
| FLT3 | DFGLARY | B4KJM3 | insects |
| FLT3 | DFGLARY | B4LSH1 | insects |
| FLT3 | DFGLARY | B4MTJ7 | insects |
| FLT3 | DFGLARY | B4NWV3 | insects |
| FLT3 | DFGLARY | D2A292 | insects |
| FLT3 | DFGLARY | E0VDK7 | insects |
| FLT3 | DFGLARY | E2A951 | insects |
| FLT3 | DFGLARY | E2BJ82 | insects |
| FLT3 | DFGLARY | G6DKF6 | insects |
| FLT3 | DFGLARY | H9IUP4 | insects |
| FLT3 | DFGLARY | J9K931 | insects |
| FLT3 | DFGLARY | K7IYU7 | insects |
| FLT3 | DFGLARY | Q16L66 | insects |
| FLT3 | DFGLARY | Q17AT5 | insects |
| FLT3 | DFGLARY | Q7PNL8 | insects |
| FLT3 | DFGLARY | Q7Q4A9 | insects |
| FLT3 | DFGLARY | T1GGI8 | insects |
| FLT3 | DFGLARY | U4UI89 | insects |
| FLT3 | DFGLARY | V9IMN4 | insects |
| FLT3 | DFGLARY | W5J1S8 | insects |
| FLT3 | DFGLARY | W5J5I5 | insects |
| FLT3 | DFGLARY | W8AU04 | insects |
| FLT3 | DFGLARY | W8AU08 | insects |
| FLT3 | DFGLARY | W8BGB8 | insects |
| FLT3 | DFGLARY | W8BSY6 | insects |
| RET | PVKWT | A0A026W269 | insects |
| PDGFRA | ICDFGLARV | A0A026W359 | insects |
| PDGFRA | ICDFGLARV | A0A026WAQ7 | insects |
| PDGFRA | ICDFGLARV | A0A034VNH7 | insects |
| PDGFRA | ICDFGLARV | A0A034W3U1 | insects |
| PDGFRA | ICDFGLARV | A0A088AJ01 | insects |
| RET | PVKWT | A0A0R3P952 | insects |
| PDGFRA | ICDFGLARV | B0WD06 | insects |
| PDGFRA | ICDFGLARV | B0WDB3 | insects |
| RET | PVKWT | B0WKZ9 | insects |
| PDGFRA | ICDFGLARV | B3M5P1 | insects |
| RET | PVKWT | B3M9I4 | insects |
| PDGFRA | ICDFGLARV | B3NF68 | insects |
| RET | PVKWT | B4IZ80 | insects |
| PDGFRA | DFGLARV | B4JLN7 | insects |
| PDGFRA | ICDFGLARV | B4KUQ0 | insects |
| RET | PVKWT | B4KX98 | insects |
| PDGFRA | DFGLARV | B4L7Q8 | insects |
| PDGFRA | ICDFGLARV | B4LHS8 | insects |
| PDGFRA | DFGLARV | B4M6V2 | insects |
| RET | PVKWT | B4MFY6 | insects |
| RET | PVKWT | B4MMY8 | insects |
| PDGFRA | ICDFGLARV | B4N5J3 | insects |
| RET | PVKWT | B4PI98 | insects |
| RET | PVKWT | D6WH54 | insects |
| PDGFRA | ICDFGLARV | D6WZH1 | insects |
| PDGFRA | ICDFGLARV | D6X2Z2 | insects |
| PDGFRA | ICDFGLARV | E0VIW1 | insects |
| PDGFRA | ICDFGLARV | E1ZZM3 | insects |
| RET | PVKWT | E2AGB2 | insects |
| RET | PVKWT | E2B6N7 | insects |
| PDGFRA | ICDFGLARV | E2BZZ2 | insects |
| PDGFRA | DFGLARV | E2C799 | insects |
| RET | PVKWT | F4W469 | insects |
| PDGFRA | ICDFGLARV | F8QQF1 | insects |
| PDGFRA | ICDFGLARV | G6CKV7 | insects |
| PDGFRA | ICDFGLARV | G6DBH2 | insects |
| RET | PVKWT | G6DH12 | insects |
| PDGFRA | ICDFGLARV | H9JB30 | insects |
| RET | PVKWT | H9JRD4 | insects |
| PDGFRA | ICDFGLARV | J9JQB4 | insects |
| PDGFRA | ICDFGLARV | J9JTB4 | insects |
| PDGFRA | ICDFGLARV | K7IV71 | insects |
| PDGFRA | ICDFGLARV | Q16HR0 | insects |
| PDGFRA | ICDFGLARV | Q170B9 | insects |
| PDGFRA | ICDFGLARV | Q17BW3 | insects |
| RET | PVKWT | Q17CE8 | insects |
| PDGFRA | ICDFGLARV | Q23993 | insects |
| PDGFRA | ICDFGLARV | Q23994 | insects |
| PDGFRA | ICDFGLARV | Q3C2L4 | insects |
| PDGFRA | ICDFGLARV | Q5W1K9 | insects |
| PDGFRA | ICDFGLARV | Q7PNN1 | insects |
| RET | PVKWT | Q7Q5D3 | insects |
| PDGFRA | ICDFGLARV | Q8IQ91 | insects |
| PDGFRA | DFGLARV | R4FNW5 | insects |
| PDGFRA | DFGLARV | T1GI54 | insects |
| PDGFRA | ICDFGLARV | T1H1J2 | insects |
| RET | PVKWT | T1I1G4 | insects |
| PDGFRA | ICDFGLARV | T1IFJ4 | insects |
| PDGFRA | ICDFGLARV | T1PAX6 | insects |
| PDGFRA | ICDFGLARV | U4UGP1 | insects |
| PDGFRA | ICDFGLARV | V5GWA5 | insects |
| PDGFRA | ICDFGLARV | V5I8E7 | insects |
| PDGFRA | ICDFGLARV | W5J4H3 | insects |
| PDGFRA | ICDFGLARV | W5J9V7 | insects |
| RET | PVKWT | W5JAG1 | insects |
| RET | PVKWT | X2JCI2 | insects |
| KLF4 | YTQSS | B4LYX5 | insects |
| MAP2K1 | SYIVG | H9J7L7 | insects |
| RET | IPVKWT | A0A034V6J5 | insects |
| RET | IPVKWT | A0A034VBK9 | insects |
| RET | IPVKWT | A0A0P8XXZ8 | insects |
| RET | IPVKWT | B4GP50 | insects |
| RET | IPVKWT | B4HKF0 | insects |
| RET | IPVKWT | B4JI59 | insects |
| RET | IPVKWT | B4K4Y8 | insects |
| RET | IPVKWT | B4LW11 | insects |
| RET | IPVKWT | B4NAI9 | insects |
| RET | IPVKWT | B4PUM4 | insects |
| RET | IPVKWT | B4QWN6 | insects |
| RET | PVKWT | E0VM96 | insects |
| RET | PVKWT | J9JNW3 | insects |
| RET | IPVKWT | P18106 | insects |
| RET | IPVKWT | Q29BL5 | insects |
| RET | IPVKWT | T1P8M7 | insects |
| RET | IPVKWT | W8ATK3 | insects |
| RET | IPVKWT | W8BFW6 | insects |
| ABL1 | IEFM | E2R7J5 | mammals |
| ABL1 | IEFM | F1N780 | mammals |
| ABL1 | IEFM | F1RY15 | mammals |
| ABL1 | IEFM | F6Z105 | mammals |
| ABL1 | IEFM | G1P4L7 | mammals |
| ABL1 | IEFM | G1RSQ5 | mammals |
| ABL1 | IEFM | G3QUG2 | mammals |
| ABL1 | IEFM | G3TK41 | mammals |
| ABL1 | IEFM | H0WJP9 | mammals |
| ABL1 | IEFM | H2PJU4 | mammals |
| ABL1 | IEFM | H2QTF1 | mammals |
| ABL1 | IEFM | L5JPL3 | mammals |
| ABL1 | IEFM | L8IHD3 | mammals |
| ABL1 | IEFM | M3WPK6 | mammals |
| ABL1 | IEFM | M3YCD3 | mammals |
| ABL1 | IEFM | P54759 | mammals |
| ABL1 | IEFM | Q61772 | mammals |
| ABL1 | IEFM | S7PSE9 | mammals |
| ABL1 | IEFM | S9Y4L2 | mammals |
| ABL1 | IEFM | U3DW98 | mammals |
| ABL1 | IEFM | W5PRL0 | mammals |
| ALK | KIGDFGMAQ | B6D4Y8 | mammals |
| BRAF | DFGLATE | F7HBH1 | mammals |
| EGFR | DFGR | I3LUX8 | mammals |
| EGFR | DFGR | Q8BFX6 | mammals |
| FLT3 | DFGLARY | F6QXR4 | mammals |
| PDGFRA | DFGLARV | A8K379 | mammals |
| PDGFRA | DFGLARV | A8K4G3 | mammals |
| PDGFRA | DFGLARV | A8K6P8 | mammals |
| RET | PVKWT | B2R8C2 | mammals |
| PDGFRA | DFGLARV | B4DFP8 | mammals |
| PDGFRA | DFGLARV | B4DQ79 | mammals |
| PDGFRA | DFGLARV | C5ISA0 | mammals |
| PDGFRA | DFGLARV | D3K5N0 | mammals |
| PDGFRA | DFGLARV | D4QGC1 | mammals |
| PDGFRA | DFGLARV | D6RVQ8 | mammals |
| RET | PVKWT | E1BPI3 | mammals |
| PDGFRA | DFGLARV | E2RBB8 | mammals |
| PDGFRA | DFGLARV | F1PPV7 | mammals |
| RET | PVKWT | F1PR52 | mammals |
| PDGFRA | DFGLARV | F1PSI2 | mammals |
| PDGFRA | ICDFGLARV | F1RJ29 | mammals |
| PDGFRA | ICDFGLARV | F1RL02 | mammals |
| PDGFRA | DFGLARV | F1RT53 | mammals |
| RET | PVKWT | F1RZK6 | mammals |
| PDGFRA | DFGLARV | F1SI00 | mammals |
| PDGFRA | ICDFGLARV | F6RE45 | mammals |
| RET | PVKWT | F6RRJ4 | mammals |
| PDGFRA | DFGLARV | F6S1M3 | mammals |
| PDGFRA | ICDFGLARV | F6W1T7 | mammals |
| PDGFRA | DFGLARV | F6XM60 | mammals |
| PDGFRA | DFGLARV | F6Y2P0 | mammals |
| RET | PVKWT | F6Z7T8 | mammals |
| PDGFRA | ICDFGLARV | F7AK94 | mammals |
| PDGFRA | DFGLARV | F7AQZ6 | mammals |
| PDGFRA | DFGLARV | F7AT17 | mammals |
| PDGFRA | DFGLARV | F7B7A6 | mammals |
| PDGFRA | DFGLARV | F7BPR6 | mammals |
| PDGFRA | DFGLARV | F7BV42 | mammals |
| PDGFRA | DFGLARV | F7CRU9 | mammals |
| PDGFRA | DFGLARV | F7CRW3 | mammals |
| PDGFRA | DFGLARV | F7CRX1 | mammals |
| PDGFRA | ICDFGLARV | F7E823 | mammals |
| PDGFRA | ICDFGLARV | F7FHD1 | mammals |
| RET | PVKWT | F7G2C9 | mammals |
| PDGFRA | DFGLARV | F7GL84 | mammals |
| PDGFRA | DFGLARV | F7GL90 | mammals |
| RET | PVKWT | F7HTM9 | mammals |
| RET | PVKWT | F7HTW0 | mammals |
| PDGFRA | DFGLARV | F7I892 | mammals |
| PDGFRA | ICDFGLARV | F7IFD7 | mammals |
| PDGFRA | DFGLARV | G1L4Q2 | mammals |
| PDGFRA | DFGLARV | G1L994 | mammals |
| PDGFRA | DFGLARV | G1P7G0 | mammals |
| RET | PVKWT | G1PHB8 | mammals |
| PDGFRA | DFGLARV | G1QKJ0 | mammals |
| RET | PVKWT | G1RRJ9 | mammals |
| RET | PVKWT | G1SEX4 | mammals |
| PDGFRA | DFGLARV | G1SIY6 | mammals |
| PDGFRA | DFGLARV | G1STR2 | mammals |
| PDGFRA | DFGLARV | G1SXU8 | mammals |
| PDGFRA | ICDFGLARV | G1T5Y1 | mammals |
| PDGFRA | DFGLARV | G2HE11 | mammals |
| PDGFRA | DFGLARV | G3QK47 | mammals |
| PDGFRA | ICDFGLARV | G3R4R6 | mammals |
| PDGFRA | DFGLARV | G3RS83 | mammals |
| PDGFRA | ICDFGLARV | G3S5N7 | mammals |
| PDGFRA | DFGLARV | G3SGW0 | mammals |
| PDGFRA | DFGLARV | G3SIX7 | mammals |
| RET | PVKWT | G3SKL2 | mammals |
| RET | PVKWT | G3SWI8 | mammals |
| PDGFRA | DFGLARV | G3SX99 | mammals |
| PDGFRA | DFGLARV | G3T0P1 | mammals |
| PDGFRA | ICDFGLARV | G3TC07 | mammals |
| PDGFRA | ICDFGLARV | G3W0G0 | mammals |
| PDGFRA | ICDFGLARV | G3WDU3 | mammals |
| RET | PVKWT | G3X1Y4 | mammals |
| PDGFRA | DFGLARV | G7NGG0 | mammals |
| RET | PVKWT | G7P3L9 | mammals |
| PDGFRA | ICDFGLARV | G7PHB5 | mammals |
| PDGFRA | ICDFGLARV | H0WMJ7 | mammals |
| PDGFRA | DFGLARV | H0WZ35 | mammals |
| PDGFRA | ICDFGLARV | H0WZV9 | mammals |
| RET | PVKWT | H0WZZ0 | mammals |
| PDGFRA | DFGLARV | H0Y3C5 | mammals |
| PDGFRA | ICDFGLARV | H2NT22 | mammals |
| PDGFRA | ICDFGLARV | H2P4Z2 | mammals |
| RET | PVKWT | H2PK43 | mammals |
| PDGFRA | DFGLARV | H2PQC0 | mammals |
| PDGFRA | DFGLARV | H2QK59 | mammals |
| RET | PVKWT | H2QTL3 | mammals |
| PDGFRA | DFGLARV | I0FKQ7 | mammals |
| PDGFRA | ICDFGLARV | I3LCS8 | mammals |
| PDGFRA | DFGLARV | J3KPD6 | mammals |
| PDGFRA | DFGLARV | K6ZXI1 | mammals |
| PDGFRA | DFGLARV | K7CGC1 | mammals |
| PDGFRA | ICDFGLARV | K7EBP8 | mammals |
| PDGFRA | DFGLARV | K9IVM1 | mammals |
| RET | PVKWT | L5JPH6 | mammals |
| PDGFRA | DFGLARV | L5JV02 | mammals |
| PDGFRA | DFGLARV | L5K960 | mammals |
| PDGFRA | ICDFGLARV | L5KWL7 | mammals |
| PDGFRA | ICDFGLARV | L5LK62 | mammals |
| PDGFRA | ICDFGLARV | L5LN45 | mammals |
| PDGFRA | DFGLARV | L5LNS5 | mammals |
| PDGFRA | DFGLARV | L5LTI4 | mammals |
| RET | PVKWT | L5M3R8 | mammals |
| PDGFRA | ICDFGLARV | L8HUM3 | mammals |
| RET | PVKWT | L8HVF8 | mammals |
| PDGFRA | DFGLARV | L8I295 | mammals |
| PDGFRA | DFGLARV | L8IWY1 | mammals |
| RET | PVKWT | L8YBJ7 | mammals |
| PDGFRA | DFGLARV | L9KGM8 | mammals |
| PDGFRA | DFGLARV | L9LAK6 | mammals |
| PDGFRA | DFGLARV | M1JPB0 | mammals |
| PDGFRA | DFGLARV | M1KQ50 | mammals |
| PDGFRA | DFGLARV | M3VXI2 | mammals |
| RET | PVKWT | M3W1G7 | mammals |
| PDGFRA | DFGLARV | M3W7A8 | mammals |
| PDGFRA | ICDFGLARV | M3XVL2 | mammals |
| PDGFRA | DFGLARV | M3YB15 | mammals |
| RET | PVKWT | M3YFV3 | mammals |
| PDGFRA | DFGLARV | P28482 | mammals |
| PDGFRA | DFGLARV | P08631 | mammals |
| PDGFRA | DFGLARV | P25911 | mammals |
| RET | PVKWT | P42685 | mammals |
| PDGFRA | DFGLARV | Q07014 | mammals |
| PDGFRA | ICDFGLARV | Q499G7 | mammals |
| PDGFRA | DFGLARV | Q4KM34 | mammals |
| RET | PVKWT | Q4R6L8 | mammals |
| PDGFRA | DFGLARV | Q9UBE8 | mammals |
| PDGFRA | DFGLARV | Q5EDC3 | mammals |
| PDGFRA | DFGLARV | Q8IZL9 | mammals |
| PDGFRA | DFGLARV | Q5R7I7 | mammals |
| RET | PVKWT | Q62662 | mammals |
| PDGFRA | DFGLARV | Q8IZL9 | mammals |
| RET | PVKWT | Q922K9 | mammals |
| PDGFRA | DFGLARV | Q9JHU3 | mammals |
| PDGFRA | DFGLARV | S7MJJ3 | mammals |
| PDGFRA | ICDFGLARV | S7NB02 | mammals |
| RET | PVKWT | S7NLX8 | mammals |
| PDGFRA | ICDFGLARV | S7PU27 | mammals |
| PDGFRA | DFGLARV | S7Q5R3 | mammals |
| PDGFRA | DFGLARV | S9WDH1 | mammals |
| RET | PVKWT | S9WDW3 | mammals |
| PDGFRA | DFGLARV | S9X9E4 | mammals |
| PDGFRA | DFGLARV | S9XFB1 | mammals |
| PDGFRA | ICDFGLARV | S9XG55 | mammals |
| PDGFRA | DFGLARV | U3CC14 | mammals |
| PDGFRA | DFGLARV | U3CYN2 | mammals |
| PDGFRA | ICDFGLARV | U3CZ42 | mammals |
| PDGFRA | DFGLARV | U3D0I6 | mammals |
| PDGFRA | DFGLARV | U3D9D3 | mammals |
| PDGFRA | ICDFGLARV | U3DQA9 | mammals |
| PDGFRA | DFGLARV | U3EBU1 | mammals |
| PDGFRA | DFGLARV | W5NXU7 | mammals |
| PDGFRA | DFGLARV | W5NXU9 | mammals |
| RET | PVKWT | W5PI49 | mammals |
| KLF4 | QSSHL | F6S429 | mammals |
| KLF4 | QSSHL | G3WK25 | mammals |
| RET | PVKWT | A8K3B6 | mammals |
| RET | PVKWT | B2RDR6 | mammals |
| RET | PVKWT | B4DP99 | mammals |
| RET | PVKWT | B4DUD9 | mammals |
| RET | PVKWT | B5BU52 | mammals |
| RET | PVKWT | E7ENM8 | mammals |
| RET | PVKWT | F1MD57 | mammals |
| RET | IPVKWT | F1N1Q0 | mammals |
| RET | IPVKWT | F1PEM3 | mammals |
| RET | PVKWT | F1PUK7 | mammals |
| RET | PVKWT | F1Q093 | mammals |
| RET | PVKWT | F1RMJ0 | mammals |
| RET | PVKWT | F1S7P3 | mammals |
| RET | PVKWT | F1SJ27 | mammals |
| RET | PVKWT | F6QS08 | mammals |
| RET | PVKWT | F6RWR2 | mammals |
| RET | PVKWT | F6SUV2 | mammals |
| RET | PVKWT | F6U5B0 | mammals |
| RET | PVKWT | F6W9M0 | mammals |
| RET | IPVKWT | F6XJB6 | mammals |
| RET | PVKWT | F6XQG4 | mammals |
| RET | PVKWT | F6Y8A0 | mammals |
| RET | PVKWT | F6ZJQ5 | mammals |
| RET | PVKWT | F6ZZA7 | mammals |
| RET | IPVKWT | F7A1L4 | mammals |
| RET | PVKWT | F7B6M0 | mammals |
| RET | IPVKWT | F7BQU5 | mammals |
| RET | IPVKWT | F7C2C5 | mammals |
| RET | IPVKWT | F7FMT3 | mammals |
| RET | PVKWT | F7GJZ7 | mammals |
| RET | PVKWT | F7GP25 | mammals |
| RET | PVKWT | F7GRP2 | mammals |
| RET | PVKWT | F7GU57 | mammals |
| RET | IPVKWT | F7H0I3 | mammals |
| RET | IPVKWT | F7H0J0 | mammals |
| RET | PVKWT | F7IBY8 | mammals |
| RET | PVKWT | F7IC12 | mammals |
| RET | PVKWT | F7IEC5 | mammals |
| RET | PVKWT | G1M5C6 | mammals |
| RET | PVKWT | G1PN95 | mammals |
| RET | PVKWT | G1QRD1 | mammals |
| RET | IPVKWT | G1RGE0 | mammals |
| RET | PVKWT | G1RNK7 | mammals |
| RET | PVKWT | G1RWZ7 | mammals |
| RET | PVKWT | G1SX69 | mammals |
| RET | PVKWT | G3QYQ6 | mammals |
| RET | IPVKWT | G3R6S9 | mammals |
| RET | PVKWT | G3RFH4 | mammals |
| RET | PVKWT | G3SX96 | mammals |
| RET | PVKWT | G3T005 | mammals |
| RET | PVKWT | G3TUJ7 | mammals |
| RET | PVKWT | G3W6K1 | mammals |
| RET | PVKWT | G3WSI5 | mammals |
| RET | IPVKWT | G3X1L2 | mammals |
| RET | PVKWT | G7MVY9 | mammals |
| RET | PVKWT | G7MY70 | mammals |
| RET | PVKWT | G7NLV5 | mammals |
| RET | PVKWT | G7P9G8 | mammals |
| RET | PVKWT | G7PYK8 | mammals |
| RET | PVKWT | H0WPT7 | mammals |
| RET | PVKWT | H0WSD9 | mammals |
| RET | PVKWT | H2NNS6 | mammals |
| RET | PVKWT | H2NP74 | mammals |
| RET | PVKWT | H2NX11 | mammals |
| RET | IPVKWT | H2P2M2 | mammals |
| RET | PVKWT | H2Q9T1 | mammals |
| RET | PVKWT | H2QA35 | mammals |
| RET | PVKWT | H2QEZ8 | mammals |
| RET | IPVKWT | H2QKS8 | mammals |
| RET | PVKWT | H2R1J1 | mammals |
| RET | PVKWT | H9H312 | mammals |
| RET | PVKWT | K7CQE2 | mammals |
| RET | PVKWT | K7EQY5 | mammals |
| RET | IPVKWT | L5JY97 | mammals |
| RET | PVKWT | L5JZ33 | mammals |
| RET | PVKWT | L5L4T7 | mammals |
| RET | PVKWT | L5L6Q3 | mammals |
| RET | PVKWT | L5LIE4 | mammals |
| RET | IPVKWT | L5LJL7 | mammals |
| RET | PVKWT | L5MH88 | mammals |
| RET | PVKWT | L5MIA7 | mammals |
| RET | PVKWT | L8INW4 | mammals |
| RET | PVKWT | L8XYZ9 | mammals |
| RET | PVKWT | L9KJZ6 | mammals |
| RET | PVKWT | M3VUK8 | mammals |
| RET | PVKWT | M3W596 | mammals |
| RET | IPVKWT | M3WA08 | mammals |
| RET | PVKWT | M3WXI7 | mammals |
| RET | PVKWT | M3XYI5 | mammals |
| RET | IPVKWT | M3YAC9 | mammals |
| RET | PVKWT | M3YW42 | mammals |
| RET | PVKWT | P07332 | mammals |
| RET | PVKWT | P14238 | mammals |
| RET | PVKWT | P32577 | mammals |
| RET | PVKWT | P41241 | mammals |
| RET | PVKWT | P41242 | mammals |
| RET | PVKWT | P41243 | mammals |
| RET | PVKWT | P42679 | mammals |
| RET | PVKWT | Q0VBZ0 | mammals |
| RET | PVKWT | Q16176 | mammals |
| RET | PVKWT | Q2VXS9 | mammals |
| RET | PVKWT | Q58D16 | mammals |
| RET | PVKWT | Q5E9H3 | mammals |
| RET | IPVKWT | Q62270 | mammals |
| RET | IPVKWT | Q9H3Y6 | mammals |
| RET | PVKWT | S7MG42 | mammals |
| RET | PVKWT | S7QAG6 | mammals |
| RET | PVKWT | S9WA13 | mammals |
| RET | IPVKWT | S9YKF2 | mammals |
| RET | PVKWT | T0NJ47 | mammals |
| RET | PVKWT | T0NKK0 | mammals |
| RET | PVKWT | U3CTN9 | mammals |
| RET | PVKWT | U3D190 | mammals |
| RET | PVKWT | U3EBF2 | mammals |
| RET | PVKWT | U3ELX4 | mammals |
| RET | PVKWT | U3F8E0 | mammals |
| RET | PVKWT | W5NZ63 | mammals |
| RET | IPVKWT | W5PM85 | mammals |
| RET | PVKWT | W5PPY4 | mammals |
| RET | PVKWT | W5PSX7 | mammals |
| ABL1 | IEFM | A0A024U2T9 | protists |
| ABL1 | IEFM | A0A024UTA3 | protists |
| ABL1 | IEFM | A0A024UUY8 | protists |
| ABL1 | IEFM | A0A0A1U0K8 | protists |
| ABL1 | YIII | A0BFV6 | protists |
| ABL1 | YIII | A0BQQ8 | protists |
| ABL1 | IIIEF | A2EA15 | protists |
| ABL1 | YIII | A2ESM2 | protists |
| ABL1 | IEFM | A9VA36 | protists |
| ABL1 | IIIEF | B0EJH3 | protists |
| ABL1 | IEFM | D0MVJ0 | protists |
| ABL1 | IEFM | F2UKB6 | protists |
| ABL1 | IEFM | H3GYV8 | protists |
| ABL1 | YIII | I7MKK8 | protists |
| ABL1 | IIIEF | K2HMF6 | protists |
| ABL1 | IEFM | L8GHZ9 | protists |
| ABL1 | IIIEF | M7WI41 | protists |
| ABL1 | IEFM | V9FVN8 | protists |
| ABL1 | IEFM | V9FVS3 | protists |
| ABL1 | IEFM | W2FNL0 | protists |
| ABL1 | IEFM | W2K384 | protists |
| ABL1 | IEFM | W2PIU8 | protists |
| ABL1 | IEFM | W2QR72 | protists |
| ABL1 | IEFM | W2QRM4 | protists |
| ABL1 | IEFM | W2QRM8 | protists |
| ABL1 | IEFM | W2VU38 | protists |
| ABL1 | IEFM | W2Y4F6 | protists |
| AKT1 | KRGK | A2DNC5 | protists |
| BRAF | GDFGLATE | A0CA48 | protists |
| BRAF | GDFGLATE | A0CEN2 | protists |
| BRAF | IGDFGLATE | D2V521 | protists |
| BRAF | IGDFGLATE | D2VZP4 | protists |
| BRAF | GDFGLATE | D8M5N7 | protists |
| BRAF | EKSR | F0ZSC9 | protists |
| BRAF | EKSR | P18161 | protists |
| EGFR | DFGR | F2UIW9 | protists |
| EGFR | DFGR | G5A4H8 | protists |
| EGFR | DFGR | L8HHT8 | protists |
| ERBB2 | VAIKVS | A0E1N8 | protists |
| ERBB2 | AIKVS | A2GDT3 | protists |
| ERBB2 | VAIKVS | G0TUM2 | protists |
| ERBB2 | AIKVS | I7LV96 | protists |
| ERBB2 | AIKVS | I7M8C4 | protists |
| FLT3 | DFGLARY | A0A023B478 | protists |
| FLT3 | ICDFGLARY | A2EBK6 | protists |
| FLT3 | DFGLARY | A3FQD3 | protists |
| FLT3 | DFGLARY | B6ADS2 | protists |
| FLT3 | DFGLARY | F2TVW2 | protists |
| FLT3 | ICDFGLARY | J9I5I7 | protists |
| FLT3 | CDFGLARY | J9IU28 | protists |
| FLT3 | DFGLARY | Q4XWF8 | protists |
| FLT3 | DFGLARY | W7AYN7 | protists |
| FLT3 | DFGLARY | W7TXV2 | protists |
| PDGFRA | DFGLARV | A0A024FUI5 | protists |
| PDGFRA | DFGLARV | A0A024G3J6 | protists |
| PDGFRA | DFGLARV | A0A024G3K0 | protists |
| PDGFRA | DFGLARV | A0A024GAR5 | protists |
| PDGFRA | ICDFGLARV | A0A024GDD5 | protists |
| PDGFRA | DFGLARV | A0A024GGT3 | protists |
| PDGFRA | DFGLARV | A0A024TY50 | protists |
| PDGFRA | DFGLARV | A0A024TYK3 | protists |
| PDGFRA | DFGLARV | A0A024TYM5 | protists |
| PDGFRA | DFGLARV | A0A024TZL9 | protists |
| PDGFRA | ICDFGLARV | A0A024UGP7 | protists |
| PDGFRA | ICDFGLARV | A0A024UL26 | protists |
| PDGFRA | ICDFGLARV | A0A024ULE2 | protists |
| PDGFRA | ICDFGLARV | A0A058ZBK5 | protists |
| PDGFRA | DFGLARV | A0A058ZEM5 | protists |
| PDGFRA | DFGLARV | A0A081A847 | protists |
| PDGFRA | DFGLARV | A0A0A1U073 | protists |
| PDGFRA | DFGLARV | A0A0W8DCD2 | protists |
| PDGFRA | GLARV | A0C2T2 | protists |
| PDGFRA | DFGLARV | A0C2Z5 | protists |
| PDGFRA | DFGLARV | A0CTK9 | protists |
| KIT | ARVIK | A0CX89 | protists |
| PDGFRA | DFGLARV | A0DIY7 | protists |
| PDGFRA | DFGLARV | A0DRH3 | protists |
| PDGFRA | DFGLARV | A2FFG0 | protists |
| PDGFRA | DFGLARV | A2FMB8 | protists |
| PDGFRA | DFGLARV | A3FPT3 | protists |
| PDGFRA | CDFGLARV | A9UQF4 | protists |
| PDGFRA | DFGLARV | A9V4U4 | protists |
| PDGFRA | ICDFGLARV | A9V8H1 | protists |
| KIT | DFGLARV | A9V9C2 | protists |
| PDGFRA | DFGLARV | A9V9Z1 | protists |
| PDGFRA | DFGLARV | A9VA07 | protists |
| PDGFRA | DFGLARV | A9VAA5 | protists |
| PDGFRA | DFGLARV | D0MYT9 | protists |
| PDGFRA | DFGLARV | D0N983 | protists |
| PDGFRA | DFGLARV | D0NUE0 | protists |
| PDGFRA | DFGLARV | D2VMC2 | protists |
| PDGFRA | DFGLARV | F0VZE9 | protists |
| PDGFRA | ICDFGLARV | F0W4U8 | protists |
| PDGFRA | DFGLARV | F0W647 | protists |
| PDGFRA | DFGLARV | F0WPQ9 | protists |
| PDGFRA | DFGLARV | F0WSU8 | protists |
| PDGFRA | DFGLARV | F0WSV0 | protists |
| PDGFRA | ICDFGLARV | F0Y0W9 | protists |
| PDGFRA | GLARV | F1A4S5 | protists |
| PDGFRA | DFGLARV | F2TY67 | protists |
| PDGFRA | DFGLARV | F2U5L0 | protists |
| PDGFRA | DFGLARV | F2U842 | protists |
| PDGFRA | CDFGLARV | F2UEG2 | protists |
| PDGFRA | DFGLARV | F2UI96 | protists |
| PDGFRA | DFGLARV | F2UKU9 | protists |
| PDGFRA | DFGLARV | F2ULL0 | protists |
| PDGFRA | ICDFGLARV | F2US32 | protists |
| PDGFRA | DFGLARV | G4YMK4 | protists |
| PDGFRA | DFGLARV | G4ZPW1 | protists |
| PDGFRA | DFGLARV | G5AA26 | protists |
| PDGFRA | DFGLARV | H3G9Y3 | protists |
| PDGFRA | DFGLARV | H3GHI1 | protists |
| PDGFRA | DFGLARV | H3HDP2 | protists |
| PDGFRA | DFGLARV | J9IHV1 | protists |
| PDGFRA | DFGLARV | J9IJC8 | protists |
| PDGFRA | DFGLARV | K3WDI0 | protists |
| PDGFRA | DFGLARV | L8H7P6 | protists |
| PDGFRA | DFGLARV | L8HA88 | protists |
| PDGFRA | DFGLARV | M4BBK8 | protists |
| PDGFRA | DFGLARV | M4BH68 | protists |
| PDGFRA | DFGLARV | M4BTV9 | protists |
| KIT | DFGLARV | M4BZ82 | protists |
| PDGFRA | DFGLARV | R1CCX2 | protists |
| KIT | DFGLARV | S9UTH5 | protists |
| PDGFRA | DFGLARV | S9VPV3 | protists |
| PDGFRA | DFGLARV | S9VZD2 | protists |
| PDGFRA | DFGLARV | T0Q3T4 | protists |
| PDGFRA | ICDFGLARV | T0Q8Y3 | protists |
| PDGFRA | ICDFGLARV | T0RPD9 | protists |
| PDGFRA | ICDFGLARV | T0RRU9 | protists |
| PDGFRA | DFGLARV | W2J059 | protists |
| PDGFRA | DFGLARV | W2KJ21 | protists |
| PDGFRA | DFGLARV | W2L7T2 | protists |
| PDGFRA | DFGLARV | W2MPW0 | protists |
| PDGFRA | DFGLARV | W2QLM3 | protists |
| PDGFRA | DFGLARV | W2WC89 | protists |
| PDGFRA | ICDFGLARV | W4FM38 | protists |
| PDGFRA | ICDFGLARV | W4FM78 | protists |
| PDGFRA | ICDFGLARV | W4FNQ7 | protists |
| PDGFRA | DFGLARV | W4FWG9 | protists |
| PDGFRA | ICDFGLARV | W4H3F2 | protists |
| PDGFRA | ICDFGLARV | W4H3T1 | protists |
| PDGFRA | ICDFGLARV | W4H4L6 | protists |
| PDGFRA | ICDFGLARV | W4H5K3 | protists |
| PDGFRA | ICDFGLARV | W4H5L7 | protists |
| PDGFRA | DFGLARV | W6L558 | protists |
| MAP2K1 | SYIV | A2G6P7 | protists |
| MAP2K1 | SYIV | B0EV44 | protists |
| MAP2K1 | SYIV | D2VK10 | protists |
| MAP2K1 | CNSS | G0QIY0 | protists |
| MAP2K1 | SYIV | K2H3B2 | protists |
| MAP2K1 | SYIV | M3TBF7 | protists |
| MAP2K1 | SYIV | T0QZL1 | protists |
| MAP2K1 | NSSYI | T0SGK4 | protists |
| RET | TAIE | F2UAL0 | protists |
| RET | TAIE | F2UBD3 | protists |
| RET | PVKWT | L8GM38 | protists |
| RET | IPVKWT | L8GQU3 | protists |
| ABL1 | IEFM | A8XUH7 | roundworms |
| ABL1 | IEFM | E3MS67 | roundworms |
| ABL1 | IEFM | G0MXY6 | roundworms |
| ABL1 | IEFM | G0PLD0 | roundworms |
| BRAF | DFGLATE | A0A016UG03 | roundworms |
| BRAF | DFGLATE | A0A016UGH3 | roundworms |
| BRAF | DFGLATE | A0A016UI18 | roundworms |
| BRAF | DFGLATE | E5SE42 | roundworms |
| BRAF | DFGLATE | H2VLK4 | roundworms |
| BRAF | DFGLATE | H3FFZ3 | roundworms |
| BRAF | DFGLATE | Q965F6 | roundworms |
| BRAF | DFGLATE | U6NGN2 | roundworms |
| BRAF | DFGLATE | U6NHI9 | roundworms |
| EGFR | FGRAK | G0NUD3 | roundworms |
| EGFR | FGRAK | G0P586 | roundworms |
| EGFR | FGRAK | G0PGR9 | roundworms |
| EGFR | FGRAK | G0PGS1 | roundworms |
| ERBB2 | VAIKVS | A8XG92 | roundworms |
| ERBB2 | IPVAIKVS | D6R8W4 | roundworms |
| ERBB2 | AIKVS | E3NAU4 | roundworms |
| ERBB2 | AIKVS | G0NWI6 | roundworms |
| ERBB2 | AIKVS | G0NWJ0 | roundworms |
| FLT3 | DFGLARY | A0A044SLR7 | roundworms |
| FLT3 | DFGLARY | F1KZ79 | roundworms |
| FLT3 | DFGLARY | J9F2L1 | roundworms |
| FLT3 | DFGLARY | U6NSL9 | roundworms |
| FLT3 | DFGLARY | W2SXZ8 | roundworms |
| PDGFRA | ICDFGLARV | A0A016RT62 | roundworms |
| KIT | CDFGLARV | A0A016RT65 | roundworms |
| KIT | ICDFGLARV | A0A016RTB3 | roundworms |
| PDGFRA | ICDFGLARV | A0A016RUC3 | roundworms |
| PDGFRA | ICDFGLARV | A0A016RUC8 | roundworms |
| PDGFRA | DFGLARV | A0A016TX34 | roundworms |
| PDGFRA | ICDFGLARV | A0A044V924 | roundworms |
| PDGFRA | ICDFGLARV | A8XZI6 | roundworms |
| PDGFRA | ICDFGLARV | D7R7W6 | roundworms |
| PDGFRA | ICDFGLARV | E3N6S0 | roundworms |
| PDGFRA | ICDFGLARV | E3N6Z0 | roundworms |
| PDGFRA | ICDFGLARV | E5SBW7 | roundworms |
| PDGFRA | ICDFGLARV | E5SCY8 | roundworms |
| PDGFRA | DFGLARV | E5STR8 | roundworms |
| PDGFRA | ICDFGLARV | F1KXH9 | roundworms |
| PDGFRA | ICDFGLARV | F1L8C6 | roundworms |
| PDGFRA | ICDFGLARV | G0MG58 | roundworms |
| PDGFRA | DFGLARV | G0MPI1 | roundworms |
| PDGFRA | DFGLARV | G0NLL9 | roundworms |
| PDGFRA | DFGLARV | H3FLR8 | roundworms |
| PDGFRA | ICDFGLARV | J9FML0 | roundworms |
| PDGFRA | ICDFGLARV | K7GZ49 | roundworms |
| PDGFRA | ICDFGLARV | P39745 | roundworms |
| PDGFRA | ICDFGLARV | Q06AJ2 | roundworms |
| PDGFRA | ICDFGLARV | Q5K4T9 | roundworms |
| PDGFRA | DFGLARV | U6PCA2 | roundworms |
| PDGFRA | ICDFGLARV | U6Q092 | roundworms |
| PDGFRA | DFGLARV | W2TBJ6 | roundworms |
| PDGFRA | ICDFGLARV | W2TD45 | roundworms |
| PDGFRA | ICDFGLARV | W2TDL8 | roundworms |

**Supplementary Table S4. Discarded mammalian onco-homologs in Class II.**

| Gene | Onco-homologs |
| --- | --- |
| BRAF | F7HBH1 |
| FLT3 | F6QXR4 |
| EGFR | I3LUX8 |
| EGFR | Q8BFX6 |

**Supplementary Table S5. Onco-homologs in protists with the highest identity (BLASTP)　with oncogenes．**

| TK oncogene | Best onco-homolog (UniProtKB) | Species |
| --- | --- | --- |
| ABL1 | Q7YZH5 | Monosiga brevicollis |
| BRAF | P18161 | Dictyostelium discoideum |
| EGFR | F2UIW9 | Salpingoeca rosetta |
| FLT3 | F2TVW2 | Salpingoeca rosetta |
| KIT/PDGFRA | F2UI96 | Salpingoeca rosetta |
| MAP2K1 | A0DBD5 | Paramecium tetraurelia |
| RET | F2UBD3 | Salpingoeca rosetta |

**Reference**

1 Finn, R. D. *et al.* The Pfam protein families database: towards a more sustainable future. *Nucleic Acids Research* **44**, D279 (2016).

2 Apweiler, R. *et al.* UniProt: the universal protein knowledgebase. *Nucleic acids research* **32**, D115-D119 (2004).

3 Altschul, S. F. *et al.* Gapped BLAST and PSI-BLAST: a new generation of protein database search programs. *Nucleic acids research* **25**, 3389-3402 (1997).
